# Editorial: Melatonin in Plants

**DOI:** 10.3389/fpls.2017.01666

**Published:** 2017-09-25

**Authors:** Haitao Shi, John Love, Wei Hu

**Affiliations:** ^1^Hainan Key Laboratory for Sustainable Utilization of Tropical Bioresources and College of Biology, Institute of Tropical Agriculture and Forestry, Hainan University Haikou, China; ^2^Biosciences, College of Life and Environmental Sciences, University of Exeter Exeter, United Kingdom; ^3^Key Laboratory of Biology and Genetic Resources of Tropical Crops, Institute of Tropical Bioscience and Biotechnology, Chinese Academy of Tropical Agricultural Sciences Haikou, China

**Keywords:** melatonin, plant, reactive oxygen species (ROS), development, stress responses

Melatonin (*N*-acetyl-5-methoxytryptamine) is an important pleiotropic molecule with multiple physiological and cellular actions in animals and plants. In 1958, melatonin was identified in the pineal gland of bovine. In 1995, melatonin was discovered in higher plants. Thereafter, the numerous functions of melatonin in animals have shown its great potential in plant physiology.

The plant melatonin field is dynamic as evidenced in the increasing number of publications in all disciplines, including its involvement in seed germination, primary root and lateral root architecture, photoprotection, circadian rhythm, flowering time, biomass production, leaf senescence and fruit ripening. Compelling evidence suggests that melatonin is also involved in various stress responses. Most of these studies indicate that melatonin may act as the first barrier in response to reactive oxygen species (ROS) burst by scavenging free radical, and as the second step for defense by regulating the expression of several stress-responsive genes.

This topic focuses on distribution, synthesis, metabolism, and the *in vivo* roles of melatonin in plants. We aim to ask whether and how melatonin functions as an important regulator during plant development and plant stress responses, and how melatonin network connects with different signaling pathways. This topic contains 3 reviews, 21 original research studies and 1 corrigendum.

The first section is the review and quantification of melatonin. Nawaz et al. and Nawaz et al. provided a review update the available information about the presence and actions of melatonin in different plant species including important crops, and highlighted the untraceutical value of melatonin-rich food crops (cereal, fruit and vegetables). Hardeland summarized the diversity of levels and multiplicity of functions of melatonin in plants, including the precursor, catabolism, isoenzymes, rate limitation and remarkable pleiotropy of melatonin biosynthetic pathway under various functional aspects, as well as the effects of melatonin on plant growth and stress response. Shi et al. highlighted the changes of endogenous melatonin levels under various stress conditions, melatonin-mediated stress responses through modulating several transcription factors, physiological mechanism, and the extensive reprogramming of transcriptome, proteome and metabolome. Erland et al. described a validated method for the quantification of melatonin, serotonin and the underlying biosynthetic precursors (tryptophan, tryptamine and *N*-acetylserotonin) in diverse plant culture systems. Ye et al. reported a simple and rapid quantification of plant endogenous melatonin by UPLC coupled with high resolution Orbitrap mass spectrometry.

The *in vivo* roles of melatonin were also revealed in different plant species in this topic. In bermudagrass, Fan et al. found that exogenous melatonin treatment alleviated cold damage by maintaining cell membrane stability, improving the process of photosystem II and increasing antioxidant enzyme activities. In cabbage and *Arabidopsis*, Zhang et al. found that melatonin improved anthocyanin accumulation and benefited cabbage growth, by increasing the expression levels of anthocyanin biosynthetic genes and ROS scavenging capacity. Similarly, genistein promotes anthocyanin synthesis in red cabbage in a light-dependent way, through directly regulating anthocyanin biosynthetic genes (Zhang et al.). Wang et al. found that high concentration of melatonin represses root meristem through modulation of both auxin synthesis and polar auxin transport in *Arabidopsis*. Consistently, Liang et al. found that melatonin regulates root architecture including both embryonic root and lateral root through modulation of auxin response in rice. In rice, Han et al. found that melatonin alleviated the inhibition of cold-mediated seedling growth by regulating anti-oxidative systems and photosystem II; and they also highlighted the dose dependent response of melatonin on plant physiological, biochemical and photosynthetic parameters. In cassava, Hu et al. found that melatonin delayed postharvest physiological deterioration (PPD) of cassava tuberous roots, through modulating ROS metabolism and transcriptomic reprogramming including metabolic-, ion homeostasis-, and enzyme activity-related genes as well as calcium signaling-, mitogen-activated protein kinase (MAPK) cascades-, and starch degradation-related pathways, etc. In rice, based on comprehensive transcriptional profiling of 11 melatonin related genes in different periods, tissues, in response to different treatments using published microarray data, Wei et al. provided new insight into the direct relation among melatonin biosynthesis and catabolic pathway, plant development, circadian rhythm, stress and defense reponses in rice. Wang et al. provided a new method for selecting and identifying bidirectional promoters and underlying regulatory regions in rice; and they also found that almost all these promoters and novel *cis*-sequences are melatonin independent. In switchgrass, Yuan et al. and Yuan et al. identified a large number of differentially expressed genes (DEGs) in the melatonin-rich switchgrass through RNA-seq, providing some clues of melatonin metabolism on transcriptome reprogramming in switchgrass. Szafrańska et al. and Szafrańska et al. found that melatonin can enhance oxidative stress tolerance in growing seedlings of *Pisum sativum* L., through regulating photosynthetic apparatus, water content, ROS accumulation and chlorophyll degradation. Jiao et al. identified the endophytic bacterium Bacillus amyloliquefaciens SB-9, which displayed high level of *in vitro* melatonin secretion as well as melatonin biosynthesis pathways. This study showed the occurrence of melatonin biosynthesis pathway in endophytic bacterial and the novel role of the endophytic bacterial in counteract the adverse effects of salt and drought stress in host plant roots. Ma et al. identified another endophytic bacterium Pseudomonas fluorescens RG11, which can transform tryptophan to melatonin and promote endogenous melatonin levels in grape roots. Ding et al. found the effect of exogenous melatonin on alleviating photoinhibition in tomato response to moderate light during chilling through accelerating non-photochemical quenching. Li et al. revealed the improved salt stress tolerance as well as photosynthesis and redox homeostasis of watermelon by exogenous melatonin treatment. Zheng et al. identified melatonin as an effective molecule to protect apple against waterlogging stress, through maintaining aerobic respiration, preserving photosynthesis and reducing oxidative damage. In *Nicotiana tabacum* L. line Bright Yellow 2 (BY-2) cell, Kobylińska, et al. found that proper dosage of melatonin increases cell proliferation and protects lead-induced cell death through inhibition of cytochrome c translocation.

We have to notice that several fundamental issues need to be resolved in the future. Besides this topic of melatonin in plants, we are looking forward to seeing more new findings.

## Author contributions

HS wrote and revised the manuscript. JL and WH provided suggestions and revised the manuscript. All authors approved the manuscript and the version to be published.

### Conflict of interest statement

The authors declare that the research was conducted in the absence of any commercial or financial relationships that could be construed as a potential conflict of interest.

